# Mesenchymal stem cells alleviate airway inflammation and emphysema in COPD through down-regulation of cyclooxygenase-2 via p38 and ERK MAPK pathways

**DOI:** 10.1038/srep08733

**Published:** 2015-03-04

**Authors:** Wen Gu, Lin Song, Xiao-Ming Li, Di Wang, Xue-Jun Guo, Wei-Guo Xu

**Affiliations:** 1 Department of Respiratory Medicine, Xinhua Hospital Affiliated to Shanghai Jiao Tong University School of Medicine, 1665 KongJiang Road, Shanghai 200092, China

## Abstract

Bone marrow-derived mesenchymal stem cells (MSCs) have been identified as one possible strategy for the treatment of chronic obstructive pulmonary disease (COPD). Our previous studies have demonstrated that MSC administration has therapeutic potential in airway inflammation and emphysema via a paracrine mechanism. We proposed that MSCs reverse the inflammatory process and restore impaired lung function through their interaction with macrophages. In our study, the rats were exposed to cigarette smoke (CS), followed by the administration of MSCs into the lungs for 5 weeks. Here we show that MSC administration alleviated airway inflammation and emphysema through the down-regulation of cyclooxygenase-2 (COX-2) and COX-2-mediated prostaglandin E2 (PGE2) production, possibly through the effect on alveolar macrophages. In vitro co-culture experiments provided evidence that MSCs down-regulated COX-2/PGE2 in macrophages through inhibition of the activation-associated phosphorylation of p38 MAPK and ERK. Our data suggest that MSCs may relieve airway inflammation and emphysema in CS-exposed rat models, through the inhibition of COX-2/PGE2 in alveolar macrophages, mediated in part by the p38 MAPK and ERK pathways. This study provides a compelling mechanism for MSC treatment in COPD, in addition to its paracrine mechanism.

Chronic obstructive pulmonary disease (COPD) is characterized by expiratory airflow limitation that is not fully reversible and is accompanied by inflammation and emphysematous destruction of the lung^1^. It is a leading cause of death worldwide, mainly due to cigarette smoking. There are no curative therapies, although present therapies, such as glucocorticosteroids, bronchodilators and oxygen therapy, alleviate airway inflammation, relieve airway obstruction and improve the quality of life^2^,^3^. However, recent advances in stem cell therapy suggest this may be useful in the treatment of diseases including COPD.

Several studies have shown that bone marrow-derived mesenchymal stem cells (MSCs) repair myocardial infarction and have an effect on limb ischemia and cerebral ischemia^4^,^5^,^6^,^7^, possibly due to their ability to differentiate into multiple lineages. However, MSCs have a potential effect on anti-inflammatory and immunomodulatory properties and gradually have been widely used in lung diseases^8^. MSC treatment was shown to ameliorate pulmonary fibrosis and pulmonary hypertension and suppress inflammation in murine models of acute lung injury (ALI)^9^,^10^,^11^. In COPD rat models, MSC treatment was shown to markedly affect pulmonary emphysema. A placebo-controlled randomized trial of MSC treatment in COPD patients proved to be safe and showed an early, significant decrease in the levels of circulating C-reactive protein (CRP)^12^. It is anticipated that MSC treatment is a novel potential therapy for pulmonary disease, particularly in COPD.

The mechanisms of MSC actions are only partly understood, and most previous studies focused on paracrine mechanisms in MSC treatment. In addition to paracrine actions, MSCs exert effects on inflammation and injury through interactions with the immune system rather than through direct actions on the lung. MSCs act as antigen-presenting cells and have been demonstrated to transfer mitochondria from MSCs to pulmonary alveoli for the first time^13^. MSCs can also ameliorate allergic airway inflammation by increasing T-regulatory cells or a Th1 phenotype^14^.

In our previous study, we observed that rMSC administration improved emphysema and pulmonary function through a paracrine action of soluble peptides, such as VEGF in COPD^15^. Next, we investigated the cell-to-cell interactions in addition to the paracrine actions in the inflammatory processes of COPD. It is well established that macrophages, which are effector cells, participate in many immunological reactions. In this study, we demonstrated that MSC treatment attenuated airway inflammation through the suppression of CS-induced cyclooxygenase-2 (COX-2) expression and COX-2-mediated prostaglandin E2 (PGE2) production in macrophages. The pathway that usually regulates COX-2 expression in inflammation involves a family of highly conserved intracellular signaling molecules, the mitogen-activated protein kinases (MAPKs). All three MAPKs, extracellular signal-regulated kinase (ERK), c-JUN N-terminal kinases (JNK) and p38, have been shown to be activated in inflammatory reactions in other species^16^. However, we do not know whether the MAPKs in the MSC treatment of COPD differ from others because of the complicated inflammatory and immune processes.

Thus, our current study was performed to investigate the following hypotheses: (1) MSC treatment ameliorates inflammation through the suppression of the COX-2/PGE2 pathway in COPD; (2) MSC treatment regulates COX-2 expression through the MAPK pathway in macrophages; and (3) a cell-to-cell mechanism exists in addition to the paracrine mechanism in MSC treatment in COPD.

## Results

### Isolation and characterization of MSCs

Mesenchymal stem cells were isolated from rat bone marrow aspirates, and the immunophenotype of the cells (CD11b/c, CD45, CD29 and CD90) was confirmed prior to experimental use. MSCs, which have a spindle fibroblast-like appearance, were plated after 5 days of proliferation and reached 80% confluence 14 days later. Flow cytometry analysis demonstrated that MSCs did not express the cell surface markers CD11b/c and CD45 but did express CD29 and CD90 (Supplementary Fig S1).

### MSC retention in lung tissues

We used a noninvasive luminescent dye to label and track the transplanted MSCs in rats. The CM-DiI dye, which localized in lung sections at 1 week post injection, gradually diminished over the following 8 weeks (Supplementary Fig S2A).

Flow cytometry analysis demonstrated that the percentage of CM-DiI-labeled cells was significantly higher at 1 week post injection and decreased over the following 8 weeks. However, CM-DiI-positive cells could still be detected at 8 weeks post injection. CM-DiI-labeled MSCs comprised 1.66% ± 0.09% of all viable cells in the lungs at 1 week, compared with 0.54% ± 0.04% at 4 weeks and 0.24% ± 0.033% at 8 weeks (Supplementary Fig S2B).

### MSC administration ameliorates cigarette smoke-induced inflammation and emphysema

H&E staining was performed to detect inflammation and emphysema in lung tissues. Sixteen weeks after exposure to CS, inflammatory cells infiltrated into the peribronchial and perivascular lung tissues in the CS-exposed group. This infiltration was ameliorated by MSC administration (Supplementary Fig S3A). We calculated the inflammation scores in the lung tissue using the Image-Pro Plus program (Medical Cybernetics). The peribronchial and perivascular inflammation scores were significantly higher in the CS-exposed group than in the sham-exposed group. MSC treatment in the CS-exposed group significantly reduced the higher peribronchial and perivascular inflammation scores, demonstrating that MSC treatment inhibited airway inflammation (Supplementary Fig S3C).

The CS-exposed group showed severe alveolar wall destruction and enlargement of the alveolar spaces even after the cessation of cigarette smoke exposure, whereas MSC administration prevented alveolar structure destruction (Supplementary Fig S3B). We quantified the enlargement of the alveolar spaces by determining the mean linear intercept (MLI) using the Image-Pro Plus program. The MLI was significantly higher in the CS-exposed group (CS, 101.6 ± 3.2 μm) than in the sham-exposed group (Sham, 63.2 ± 3.1 μm). However, MSC treatment decreased the MLI in the CS-exposed group with MSC treatment (CS + MSCs, 80.2 ± 4.5 μm). There was no significant difference between the sham-exposed group (Sham) and the sham-exposed group with MSC treatment (Sham + MSCs, 65.8 ± 2.3 μm) (Supplementary Fig S3D).

The concentrations of PGE2, interleukin-10 (IL-10), and interleukin-6 (IL-6) in the serum and bronchoalveolar lavage fluid (BAL) were determined by ELISA. The PGE2 and IL-6 levels significantly increased, whereas the IL-10 level significantly decreased in the CS-exposed group compared with that in the sham-exposed group. MSC administration reduced the PGE2 and IL-6 levels and elevated the IL-10 level in the serum and BAL (Supplementary Fig S3E–G).

### MSCs attenuate CS-induced COX-2 up-regulation and PGE2 production in lung tissue

**Figure 1:**
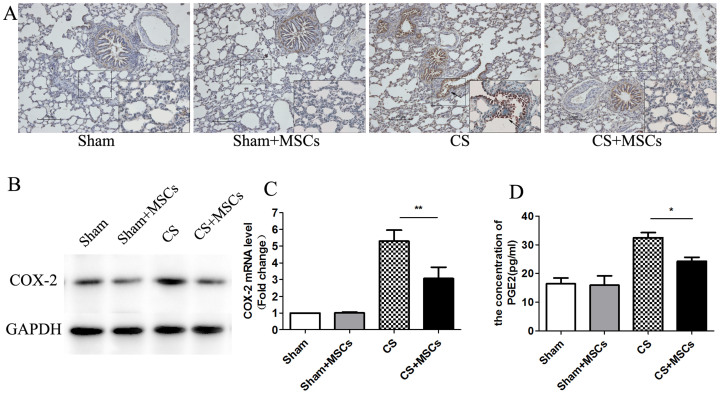
MSC administration reduced elevated COX-2 and PGE2 in lung tissue. (A). The COX-2 levels in lung tissue were measured by immunohistochemistry assay. There was a significant increase in COX-2 protein, which was localized in the bronchial epithelium and alveolar spaces, in the CS group compared with the Sham group. COX-2 expression was decreased in the CS + MSCs group compared with the CS group (n = 5, per group, ×100 magnification). (B). Lung tissues were scraped into a homogenate and analyzed by western blotting for the expression of COX-2. COX-2 expression was increased in the CS group and decreased after MSC administration. (The gels have been run under the same experimental conditions and full-length blots were presented in Supplementary Fig S4.) (C). The mRNA level of COX-2 was analyzed by real-time PCR and a significant increase in COX-2 mRNA was noted in the CS group compared with that in the Sham group. The COX-2 mRNA decreased after MSC administration. (D). Lung homogenates were collected and analyzed for PGE2 levels using a highly specific enzyme immunoassay technique. The PGE2 levels increased in the CS group and decreased in the CS + MSCs group. Data represent the mean ± SEM, n = 5, **significant difference (P < 0.01), *significant difference (P < 0.05) between the CS and CS + MSCs groups.

The up-regulation of COX-2, an inducible enzyme, has been linked to inflammatory responses, and the enzyme is believed to produce PGs involved in the inflammatory process^17^. Because our previous experiments showed an increase in PGE2 production in the serum and BAL, we investigated COX-2 expression and PGE2 synthesis in response to MSC administration in lung tissues. First, the expression of COX-2 protein in the lung tissue was determined using immunohistochemistry and was compared among the groups. COX-2 expression was increased in the alveoli, peribronchial and perivascular tissue of the CS-exposed group compared with the sham-exposed group. However, COX-2 immunoreactivity was reduced in the MSC treatment group (Fig. 1A). Second, western blot analyses confirmed higher COX-2 expression in the CS-exposed group and decreased COX-2 expression in the CS + MSCs group (Fig. 1B). Finally, quantitative PCR was performed to measure the mRNA level of COX-2. COX-2 mRNA expression was consistent with the level of COX-2 protein (Fig. 1C). We next assessed the production of PGE2, a major product of the enzymatic reaction catalyzed by COX-2, using ELISA. The PGE2 level was increased in the CS-exposed group and was reduced after treatment with MSCs, paralleling that of COX-2 (Fig. 1D).

### CD68-positive macrophages act as effector cells for the CS-induced expression of COX-2

**Figure 2:**
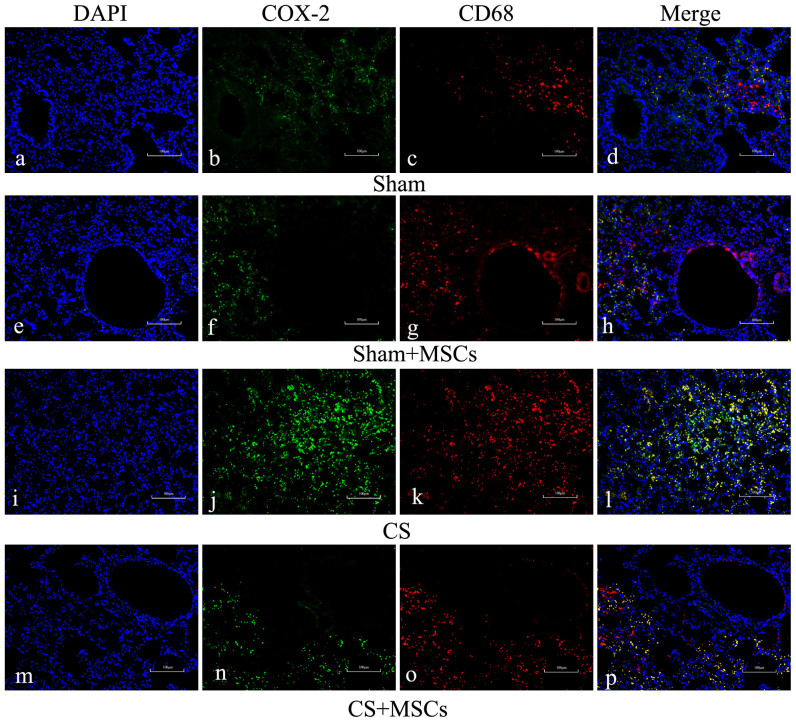
Immunohistochemistry analysis of expression and localization of COX-2 and CD68 in lung tissues. Lung sections were prepared, and immunofluorescence was used to assess the expression and subcellular localization of COX-2 (green) and CD68 (red) using a fluorescence microscopy. DAPI-staining nuclei (blue) were also shown. Images showed representative data from one of four individual experiments. Both COX-2 and CD68 expression increased in the CS group (j, k) compared with the Sham group (b, c) or Sham + MSCs group (f, g). COX-2 immunoreactivity was primarily localized in CD68-positive macrophages (l). After MSC administration, COX-2 expression decreased significantly, and CD68 expression decreased moderately (n = 5, ×100 magnification).

Because macrophages participate in many inflammatory processes and release cytokines such as COX-2/PGE2, nitric oxide synthases (NOSs), and chemokines, we asked whether macrophages might be involved in the COX-2/PGE2 pathway. To confirm the hypothesis that macrophages act as effector cells in CS-induced inflammation, we performed double immunofluorescence staining for the macrophage markers CD68 and COX-2. CD68-positive macrophages were observed in the lung sections of each group, and an increased number of CD68-positive macrophages was present in the CS-exposed group compared with the sham-exposed group. We were intrigued to find increased COX-2 expression in CD68-positive macrophage cells, indicating that macrophages play an important role in the COX-2/PGE2 inflammatory process. The number of cells containing CD68 and COX-2 colocalization was significantly greater in the CS-exposed group than in the sham-exposed group. However, with MSC administration, a significant reduction in COX-2 protein and a moderate decrease in the number of CD68-postive macrophages were observed (Fig. 2). Therefore, the increased COX-2 protein in the CS-exposed group and decreased protein after MSC administration may have been due to the effect on macrophages.

### MSC administration reduces CS-induced macrophage promotion in BAL

**Figure 3:**
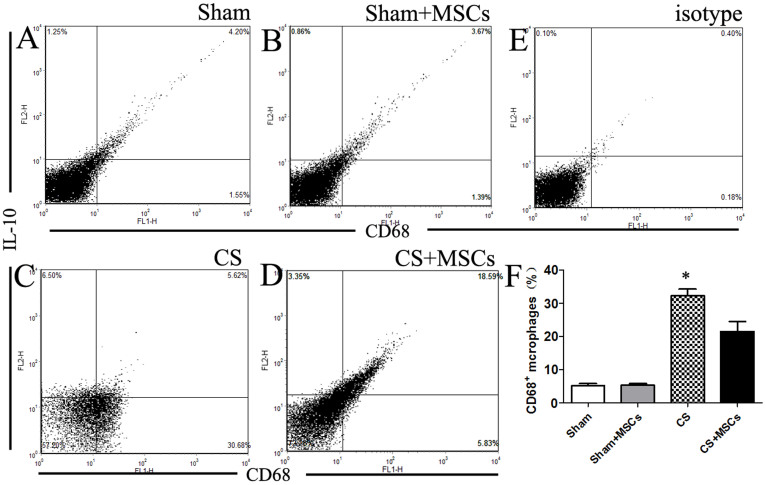
The percentage of CD68-positive cells and CD68^+^IL-10^+^ cells in BAL. (A–D). Cells isolated from BAL were stained with anti-CD68 and anti-IL-10 antibodies, and an aliquot of 10,000 cells was analyzed using flow cytometry. Cells were initially gated on forward and side scatter to remove debris and calculated using quadrant dot plots. (F). The percentage of CD68-positive cells (lower right and upper right window) in each group was plotted. The proportion of CD68-positive macrophages was significantly elevated by approximately 30% following CS treatment compared with the Sham group, and the CD68-positive cell population was reduced in the presence of MSCs. Within the CD68-positive population, the percentage of IL-10-positive (CD68^+^IL-10^+^) cells inceased by approximately 13% in the CS + MSCs group compared with the CS group. Data represent the mean ± SEM of 5 rats in each group from a single experiment and are representative of two independent experiments (n = 5) with similar results. *significant difference (P < 0.05) between the CS and CS + MSCs groups.

As we previously described, CD68-positive macrophages were reduced in lung tissue after MSC treatment. The percentage of macrophages in BAL was also analyzed. The percentage of CD68-positive macrophages was significantly elevated by approximately 30% in the CS-exposed group compared with the sham-exposed group. The percentage of CD68-positive cells in the CS-exposed group decreased by approximately 12% in response to MSC administration. Interestingly, MSC administration decreased the percentage of CD68-positive cells but increased the percentage of IL-10-positive (CD68^+^IL-10^+^) cells by 13% (Fig. 3A–F). Within the CD68-positive population, the IL-10-positive (CD68^+^IL-10^+^) cells could represent M2 macrophages. We concluded that MSC administration decreased the proportion of macrophages in the BAL of CS-exposed rat models. Furthermore, MSC administration may have increased the M2/M1 macrophage ratio in BAL.

### CS-induced COX-2 expression is reduced in macrophage cell lines co-cultured with MSCs

**Figure 4:**
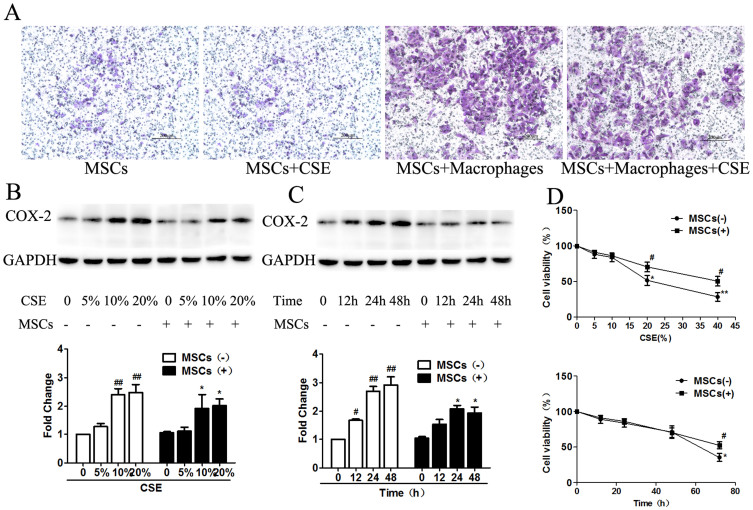
CS-induced COX-2 expression in macrophages was reduced by MSC treatment. (A). A Transwell assay was performed to analyze MSC migration. MSC migration was minimal in the absence of macrophages. However, a significant increase in cell migration occurred in MSCs co-cultured with macrophages. (B). Macrophages and MSCs were co-cultured with or without CSE solution for 12 h to 48 h at a final concentration of 5–20%, and COX-2 expression was detected using western blot analysis. MSC treatment down-regulated CS-induced COX-2 expression in a dose-dependent manner. Additionally, 10% and 20% CSE significantly induced COX-2, a result that was reversed by MSC treatment. There was no significant difference between the 10% and 20% CSE groups. (C). MSC treatment down-regulated CS-induced COX-2 expression in a time-dependent manner. CSE induced COX-2 expression at 12 h, whereas MSC treatment did not reduce COX-2 expression, likely due to an insufficient incubation time. MSC treatment reduced CS-induced COX-2 expression significantly after incubation for 24 h or 48 h. (D). The viability of macrophages was measured using the MTT assay. The cells that were incubated with 10% CSE for 48 h reached the highest cell viability. Data represent the mean ± SEM of n = 5/group; #significant difference (P < 0.05) and ##significant difference (P < 0.01) versus the corresponding untreated controls; *significant difference (P < 0.05) and **significant difference (P < 0.01) versus the corresponding MSCs(−) counterparts. (Full-length blots are presented in Supplementary Fig S5.)

To understand the effect of MSCs on macrophages, we examined the migration of MSCs in a Transwell system in vitro. In this assay, macrophages induced MSC migration, which was not eliminated by the addition of CSE to the upper chamber. A minimal amount of random migration, as shown by the number of MSCs that migrated in the absence of macrophages, was observed (Fig. 4A). These data indicated that macrophages induced MSC migration, which may have occurred in a cell-cell contact-dependent manner.

As shown in Figure 4B, CSE induced COX-2 expression in macrophages in a dose-dependent manner, whereas MSC co-culture suppressed COX-2 expression. We evaluated COX-2 protein levels in macrophages treated with increasing concentrations of CSE (5–20%). The 10% and 20% CSE groups showed a significant increase in COX-2 expression by 2- and 3-fold, respectively, compared with that in the control group, and this trend was suppressed by MSC treatment. However, there was no significant difference in COX-2 expression between the 10% CSE group and the 20% group. In addition to its dose-dependent effects, CSE stimulation also increased COX-2 expression in a time-dependent manner (Fig. 4C). CSE significantly increased COX-2 expression compared with that of the control group within 12 h. Thereafter, COX-2 levels remained elevated during the subsequent 48 h. However, there was no significant difference between the 24 h and 48 h groups. MSC treatment down-regulated COX-2 expression in all groups.

Cell survival analysis was performed using the MTT assay according to the manufacturer's instructions. We found that COX-2 expression was strikingly decreased after treatment with 20% CSE or after culture for more than 48 h. However, MSC treatment increased the viability of CS-exposed macrophages (Fig. 4D). Therefore, macrophages were exposed to 10% CSE for 48 h in the following experiments.

### MSCs attenuate CS-induced COX-2, PGE2, IL-6, and iNOS production and promote IL-10 production in macrophages

**Figure 5:**
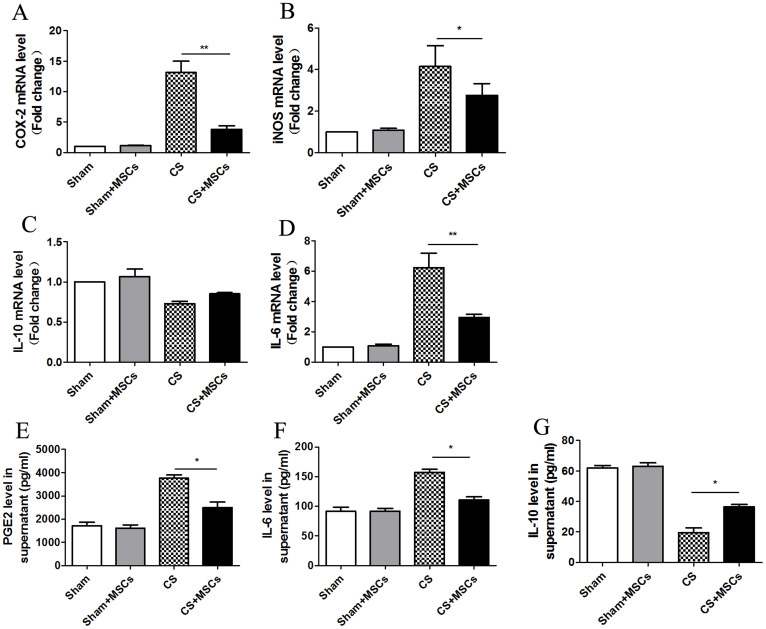
MSCs attenuated CS-induced COX-2, PGE2, IL-6, and iNOS production and promoted IL-10 production in macrophages. (A–D). Macrophages were cultured with or without MSCs in 10% CSE for 48 h and were subjected to real-time PCR analysis. CSE significantly increased the COX-2, IL-6 and iNOS mRNA expression and decreased the IL-10 mRNA expression over the vehicle control. In the MSC treatment group, the mRNA levels of COX-2, IL-6 and iNOS were decreased significantly, while the IL-10 mRNA levels increased slightly. (E–G). The culture supernatants were retained and subjected to ELISA. The PGE2 and IL-6 levels increased in supernatants and decreased after MSC treatment. However, IL-10 protein decreased in supernatants and increased after MSC treatment. Data represent the mean ± SEM of n = 5/group, *significant difference (P < 0.05), **significant difference (P < 0.01), between CS and CS + MSCs group.

Macrophages were stimulated with 10% CSE and cultured in the presence or absence of MSCs for approximately 48 h. Next, macrophages were collected and subjected to quantitative PCR to detect the mRNA levels of COX-2, IL-6, IL-10 and iNOS. CSE significantly increased the mRNA expression of COX-2, IL-6 and iNOS and decreased IL-10 mRNA expression compared with that of the Sham group. In the CS + MSCs group, the mRNA levels of COX-2, IL-6 and iNOS were decreased significantly, whereas the IL-10 mRNA levels were increased slightly (Fig. 5A–D).

The culture supernatants were retained and subjected to ELISA. We found that the PGE2 and IL-6 levels were increased significantly in the CS group and were reduced after MSC treatment. The IL-10 levels were decreased in the CS group and were increased after MSC treatment (Fig. 5E–G).

### p38 MAPK and ERK are activated in CS-stimulated macrophages and are inhibited by MSC treatment

**Figure 6:**
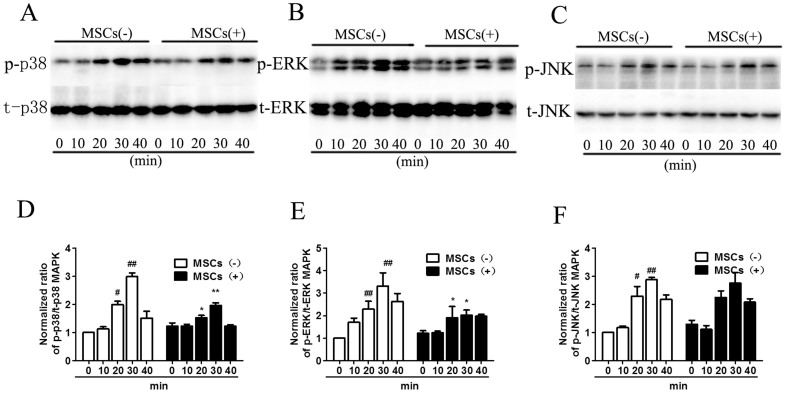
MSC treatment inhibited CS-induced phosphorylation of p38 MAPK and ERK, but not JNK in macrophages. Macrophages were treated with 10% CSE for 0–40 min, and MAPK phosphorylation was assessed in cytoplasmic extracts by western blot analysis. (A). Macrophages were probed for activation-associated phosphorylated p38 MAPK and total p38 MAPK. p38 MAPK was phosphorylated after 20 min with maximal phosphorylation at 30 min in response to CSE stimulation; the phosphorylation was inhibited by MSC treatment. (B). ERK was phosphorylated after 20 min with highest phosphorylation at 30 min in response to CSE stimulation; the phosphorylation could be inhibited by MSC treatment. (C). JNK was phosphorylated from 20 min with highest phosphorylation at 30 min in response to CSE. However, MSCs could not inhibit the activation-associated phosphorylation of JNK. Blots were representative of at least four separate experiments. Data represent the mean ± SEM, #significant difference (P < 0.05), ##significant difference (P < 0.01), versus corresponding untreated controls; *significant difference (P < 0.05), **significant difference (P < 0.01), versus corresponding MSCs(−) counterparts. (Full-length blots were presented in Supplementary Fig S6.)

The general pathway that regulates COX-2 expression during inflammation is mediated by the family of mitogen-activated protein kinases (MAPKs). It has been determined that LPS can activate all three MAP kinases, extracellular signal-regulated kinase (ERK), c-Jun N-terminal kinase (JNK) and p38. To test the hypothesis that MAPKs are essential for CSE-induced COX-2 expression, we determined whether MAPKs were activated in macrophages in response to CSE stimulation. Western blot analysis was used to determine activation-associated phosphorylation of p38 MAPK, ERK and JNK using a phospho-specific antibody. The total p38 MAPK, ERK and JNK levels were simultaneously determined to ensure that any change was due to the phosphorylation level of the protein and not disproportionate protein loading. We found that all three MAPKs were phosphorylated within 20 min and that the phosphorylation was the highest at 30 min in response to CSE stimulation in macrophages. By contrast, after MSC treatment, there was a significant decrease in p38 MAPK and ERK phosphorylation, whereas no significant difference was found in JNK phosphorylation (Fig. 6A–C). The total p38 MAPK, ERK and JNK protein levels did not change at any timepoint with or without MSC treatment. Our results showed that CSE stimulation significantly up-regulated the activation-associated phosphorylation of p38 MAPK, ERK and JNK. Additionally, MSC treatment prevented the activation of p38 MAPK and ERK but not JNK (Fig. 6D–F).

### Inhibition of p38 MAPK and ERK, but not JNK, prevents the MSC-mediated effect on CS-induced COX-2 expression in macrophages

**Figure 7:**
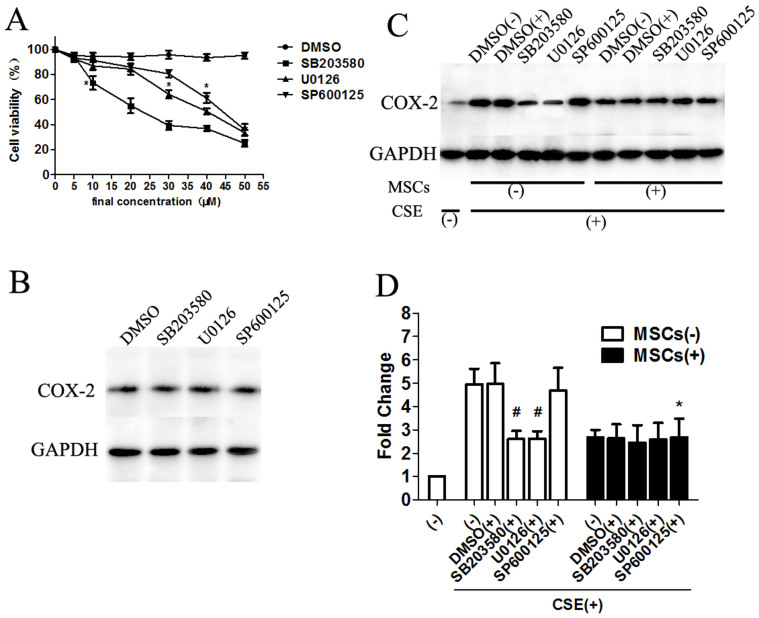
Inhibition of P38 MAPK and ERK, but not JNK prevented the MSC-mediated effect on CS-induced COX-2 expression in macrophages. Macrophages were treated with the p38 MAPK inhibitor SB203580, ERK inhibitor U0126, or JNK inhibitor SP600125, respectively, and then COX-2 protein was detected by western blot analysis after culturing with MSCs. (A). Cell viability was measured after macrophages were incubated with the three MAPK inhibitors to obtain the appropriate final concentration. Using a concentration of SB203580 above 5 μM, U0126 above 10 μM, and SP600125 above 35 μM reduced the cell viability significantly. (B). Macrophages were treated with the three MAPK inhibitors alone, and no significant differences were found in the COX-2 protein levels. (C–D). Macrophages were pretreated with SB203580 (5 μM), U0126 (10 μM), SP600125 (35 μM), or DMSO respectively, and cultured with or without MSCs. CS-induced COX-2 up-regulation could be reduced by the p38 MAPK inhibitor SB203580 and the ERK inhibitor, but not by the JNK inhibitor SP600125. After treatment with MSCs, there was no significant difference in macrophages pretreated with SB203580 or U0126. However, MSC treatment reduced COX-2 expression in macrophages treated with the JNK inhibitor SP600125, indicating that MSCs inhibited COX-2 expression via p38 MAPK and ERK signaling, but not by JNK signaling. Blots were representative of at least four separate experiments. Data represent the mean ± SEM, #significant difference (P < 0.05), versus corresponding untreated controls; *significant difference (P < 0.05), versus corresponding MSCs(−) counterparts. (Full-length blots were presented in Supplementary Fig S7.)

We subsequently investigated whether the activation of p38 MAPK and ERK plays a role in the up-regulation of COX-2 and the effect of MSC treatment on COX-2 expression using the specific p38 MAPK inhibitor SB203580, the ERK inhibitor U0126, and the JNK inhibitor SP600125. First, an MTT assay was performed to detect cell survival. The results indicated that the appropriate final concentrations of the three inhibitors had no effect on the viability of macrophages (Fig. 7A). Next, macrophages were incubated with the three MAPK inhibitors, and no significant differences were found in COX-2 protein levels, demonstrating the minimal effect of MAPK inhibitors alone on cell survival and COX-2 expression (Fig. 7B).

Finally, we determined the effects of the specific p38 MAPK inhibitor SB203580, the ERK inhibitor U0126, and the JNK inhibitor SP600125 on CSE-induced COX-2 expression. We found a significant down-regulation of COX-2 expression in CSE-stimulated macrophages treated with SB203580 and U0126. However, no statistically significant difference was observed in CSE-stimulated macrophages treated with SP600125. The usual effect observed upon MSC treatment did not occur in macrophages incubated with SB203580 or U0126. However, significantly reduced COX-2 expression was observed in cells incubated with both SP600125 and MSCs compared with those incubated with SP600125 alone. The data shown in Figure 7C indicate that MSCs reduced COX-2 expression, likely through the p38 MAPK and ERK cascades but not the JNK pathway.

### p38 MAPK and ERK signaling is required for MSC-mediated CS-induced COX-2 expression in macrophages

**Figure 8:**
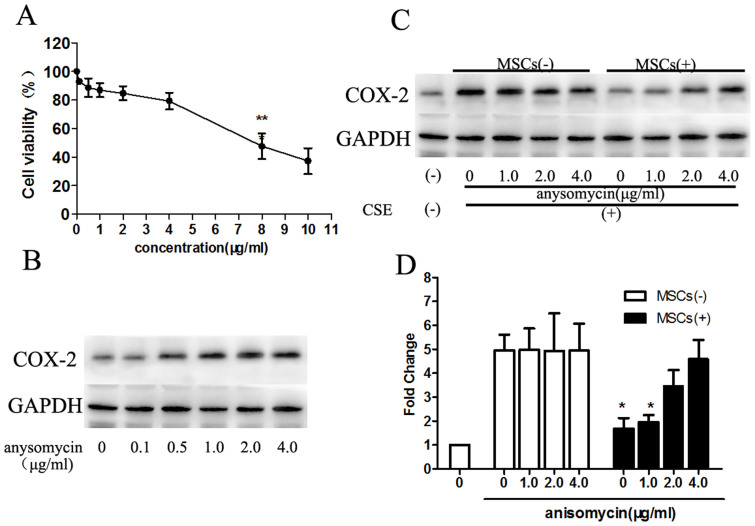
The p38 MAPK activator anisomycin induced COX-2 expression, which could be reduced partly by MSC treatment. (A). Macrophages were treated with various concentrations of anisomycin, and the cell viability was detected. The macrophage cell viability was decreased significantly above the concentration of 4 μM. (B). Anisomycin increased COX-2 expression in the concentration range of 0.1–1.0 μg/ml, but remained unchanged above the concentration of 1.0 μg/ml. (C–D). Because of the part A and B experiments, the concentrations of anisomycin at 1.0 μg/ml, 2.0 μg/ml and 4.0 μg/ml were chosen for further experiments because these levels did not affect the cell viability, as well as the COX-2 levels. MSCs reduced COX-2 expression significantly below the concentration of 1.0 μg/ml anisomycin. However, the inhibition action diminished with increased anisomycin concentrations, demonstrating that the limited MSC treatment could not absolutely reverse the effect of the p38 MAPK activator. The finding also confirmed that the MSCs down-regulated COX-2 through p38 MAPK. Blots were representative of at least four separate experiments. Data represent the mean ± SEM, *significant difference (P < 0.05), versus corresponding MSCs(−) counterparts. (Full-length blots were presented in Supplementary Fig S8.)

As described above, COX-2 expression was potently inhibited by MSC treatment, possibly due to reduced p38 MAPK or ERK phosphorylation. To confirm these observations, the role of MSC on p38 MAPK signaling in the COX-2 response was also tested using anisomycin, an activator of the p38 MAPK pathway^18^. First, macrophages were treated with different concentrations (0.1 μg/ml, 0.5 μg/ml, 1.0 μg/ml, 2.0 μg/ml, 4.0 μg/ml, 8.0 μg/ml, 10 μg/ml) of anisomycin for 6 h. MTT assays showed increased cell viability between the concentrations of 0.1 and 4.0 μg/ml. The COX-2 protein levels were increased between the concentrations of 0.1 and 1.0 μg/ml anisomycin but remained unchanged above the concentration of 1.0 μg/ml (Fig. 8A, B). Thus, we chose 1.0 μg/ml, 2.0 μg/ml and 4.0 μg/ml anisomycin for further experiments.

Following incubation with anisomycin, macrophages were treated with MSCs. As expected, different concentrations of anisomycin affected COX-2 levels in a similar manner. However, with increasing concentrations of anisomycin, the reduction in COX-2 induced by MSCs gradually decreased, indicating that MSCs could not absolutely reverse the effect of the p38 activator (Fig. 8C, D). Nonetheless, MSC treatment suppressed activated p38, demonstrating an effect on macrophages through a p38 MAPK-dependent pathway.

Next, the ERK activator C6 ceramide was used to assess ERK signaling. The results also showed a down-regulation of COX-2 mediated by MSCs through an ERK-dependent pathway (data not shown).

## Discussion

In this study, we successfully isolated MSCs and intratracheally infused the cells into CS-exposed rat models, and then examined the effects of MSC therapy and the interaction between MSCs and macrophages. Our results suggested that an intrabronchial injection of bone marrow stem cells (MSCs) was beneficial for relieving airway inflammation in CS-exposed rat models through the down-regulation of COX-2 and PGE2 synthesis.

MSC administration has been used for various inflammatory conditions, such as central nervous system injury, stroke and Crohn's disease^19^. The therapeutic potential of MSCs has also been demonstrated in several pulmonary disease models, including acute lung injury, fibrosis, pulmonary hypertention, asthma, COPD, and lung cancer. The ability of MSCs to specifically home to tumors has suggested a potential use as a delivery vehicle for cancer therapeutics^20^,^21^. Human bone marrow MSCs have been shown to suppress airway inflammation and airway hyperresponsiveness in mouse models of acute and chronic asthma^22^,^23^. In COPD rat models, bone marrow cell (BMC) treatment significantly increased cell proliferation and the number of small pulmonary vessels, reduced apoptotic cell death, and attenuated the mean pulmonary arterial pressure^24^. The therapeutic impact of autologous transplantation of adipose tissue-derived stromal cells (ASCs) has been demonstrated to enhance alveolar repair in a rat model of emphysema^25^. In this study, we showed the therapeutic effect of MSC transplantation and its possible mechanism in CS-exposed rat models that may be more related to human COPD diseases in terms of slow progression and pathological results.

In initial experiments, we labeled bone marrow-derived mesenchymal stem cells with CellTracker CM-DiI dye and visualized the cells in lung tissues after transplantation. We found that labeled MSCs persisted in the lung parenchyma 8 weeks after transplantation, demonstrating the sustained action of MSC administration. A number of studies have explored MSCs intravenously (i.v.) and discovered that cell entrapment in the lung capillary endothelium is an inevitable consequence^26^. We believe that the pulmonary first-pass effect allows the MSCs to become trapped in the lung, maintaining continuous treatment. Thus, we chose to infuse MSCs intratracheally. The outcome indicated that MSC administration attenuated airway inflammation and restored the impaired alveolar wall, as we predicted.

Although increasing data indicate that MSCs possess anti-inflammatory and immunomodulatory properties both in vitro and in vivo, their mechanism of action is still controversial^27^,^28^,^29^. Interestingly, most of these beneficial effects are thought to be mediated by the paracrine action of MSCs^30^,^31^. In our previous experiments, we found that MSC administration suppressed the inflammatory response, excessive protease expression, and cell apoptosis via a paracrine mechanism^15^. However, a paracrine mechanism cannot explain the mechanism by which those limited soluble factors have long-term immunomodulatory effects. We attempted to explore a possible cell-to-cell mechanism in addition to the paracrine mechanism by which MSCs function in CS-exposed rat models.

MSCs are capable of modulating the immune system through interactions with a wide range of immune cells. Previous studies have shown that MSCs inhibit Th2-mediated allergic airway inflammation by promoting IFN-γ and TGF-β and regulating Th cell differentiation^32^,^33^,^34^. MSCs are also known to modulate B-cell function^35^. Macrophages, which are the predominant immune effector cells, act as mediators of the inflammatory response and contribute to both the initiation and resolution of inflammation^36^. Mathias et al. elucidated that macrophages were indispensable for MSC treatment of allergic asthma, and depletion of macrophages abolished the beneficial effects of MSCs^23^. Nemeth et al. showed that MSCs attenuated sepsis by reprogramming macrophages to increase their IL-10 production^37^. Similar to their study, we showed, by intracellular staining, that the number of IL-10-producing macrophages isolated from CS-exposed rat models treated with MSCs was significantly higher. We observed that the percentage of macrophages was significantly elevated in the BAL of the CS-exposed group and was abrogated by MSC administration. Although MSCs reduced the total amount of CD68-positive macrophages, an increased proportion of IL-10-positive cells within the macrophage population was observed. Macrophages play an indispensable role in the phase of homeostasis and tissue repair and can be polarized to act as M1 or M2 macrophages during different phases of inflammation^38^,^39^. Kim et al. described a novel type of human macrophage with a potentially significant role in tissue repair after co-culture with MSCs^40^. We propose that MSCs promote an increase in M2 macrophages, which could produce an increased amount of anti-inflammatory cytokines such as IL-10. In in vitro experiments, the elevated IL-10 mRNA levels in macrophages and increased IL-10 protein in supernatants confirmed our hypothesis. Within this context, further work should be undertaken to identify a possible mechanism of M1/M2 differentiation by MSC treatment in COPD and the anti-inflammatory and immunomodulatory function of these cells.

Although previous studies have focused on MSC and macrophage interactions under diverse clinical conditions such as asthma or sepsis, those studies did not investigate the function of the interaction in COPD. Additionally, those studies primarily focused on MSCs governing proinflammatory cytokine production in macrophages. We propose for the first time that in CS-exposed rat models, infused MSCs interact with alveolar macrophages in a cell contact-dependent manner and reprogram the macrophages via the COX-2/PGE2 pathway. Our observations showed significantly increased MSC migration induced by macrophages in a Transwell assay. In parallel, it has been reported that injected BMSCs in the lung parenchyma appear to be surrounded by macrophages^37^. We and others have observed a cell-to-cell interaction between MSCs and macrophages both in vivo and in vitro.

Moreover, the central role of macrophages in COX-2 expression and related PGE2 production has been defined in other disease models, but macrophages are not involved in the mechanism governing the response to MSC administration. Thus, our study is clearly unique in several ways. We observed that COX-2 protein was primarily expressed in CD68-positive cells and was decreased after MSC treatment, clarifying the relationship between macrophages and MSC-mediated COX-2/PGE2 expression. Next, the MAPK pathway that regulates COX-2 was investigated. A family of MAPKs, including p38, ERK and JNK, was deemed to play a pivotal role in the signaling mechanism that regulates COX gene expression in the inflammatory process. However, despite extensive investigation, the pathophysiological importance of MAPKs remains unknown in MSC administration in CS-exposed rat models because of the multiple roles of MAPKs under various conditions, including inflammation and cell survival. Our study demonstrated for the first time that MSCs could reverse the activation of p38 and ERK, but not JNK, consequently leading to down-regulation of COX-2 and related cytokine production. In our experiments, MSC administration reduced COX-2 protein almost to the same degree as the p38 inhibitor SB203580 and the ERK inhibitor U0126. The use of the p38 and ERK activators verified that MSCs attenuated inflammation by down-regulating COX-2 expression through the inhibition of the activation-associated phosphorylation of p38 MAPK and ERK. It must be noted that we did not test the reason why JNK does not participate in MSC down-regulation of COX-2 or the underlying mechanism. Further elucidation of the underlying mechanisms of MAPK signaling in the macrophage response to MSCs in CS-exposed rat models will be important to understand how MSCs modify the host response to a therapeutic effect.

In summary, we demonstrate the therapeutic effect of MSC administration for CS-induced inflammation and emphysema and report for the first time that MSCs regulate COX-2 expression and PGE2 production in alveolar macrophages through p38 and ERK. Our results add to the limited studies that have investigated the mechanism of MAPK signaling in MSC treatment in CS-exposed rat models, providing hope for the further use of stem cell therapy for human COPD.

## Methods

All of the experimental protocols were approved by The Institutional Animal Care and Use Committee of Shanghai Jiaotong University. All of the methods were carried out in accordance with the Guide for the Care and Use of Laboratory Animals.

### MSC culture and characterization

Male Sprague-Dawley rats (6- to 8-weeks old; Chinese Academy of Science, Shanghai, China) were MSC donors. Under sterile conditions, the animals were anesthetized (pentobarbital 50 mg/kg intraperitoneally), the femurs and tibias were excised, the epiphyses were removed, and through needles inserted in the bone lumen, bone marrow was flushed out in a solution consisting of DMEM (Invitrogen, NY, USA) containing 10% FBS and 1% of an antibiotic mixture. The marrow was plated in tissue culture plates, and nonadherent hematopoietic cell populations were removed at day 3, followed by new media replenishment every 2 days until approximately 80% confluence. Adherent MSCs were collected and passed at low density (100–200 cells/cm^2^) and maintained in a humidified incubator (37°C; 5% CO_2_). rMSCs at passage 3–5 were harvested with 0.25% trypsin/1 mM EDTA (Life Technologies, NY, USA) for 2–3 min at 37°C and resuspended with fresh media for subsequent experiments. Next, MSCs were stained with antibodies against CD11b/c (Invitrogen, Carlsbad, CA, USA), CD45 (Invitrogen), CD29 (eBioscience, San Diego, CA, USA), and CD90 (Biolegend, San Diego, CA, USA) for flow cytometry analysis.

### Tracking CM-DiI-labeled MSCs in lung tissue

MSCs were labeled for cell retention studies with CellTracker CM-DiI (Invitrogen, Carlsbad, CA, USA) according to the manufacturer's instructions. A total of 6 × 10^6^ MSCs were resuspended in l ml Hanks' balanced salt solution, and 4 μg of CM-DiI solution was added. The cells were incubated for 30 min at 37°C, followed by 15 min at 4°C. Next, the cells were centrifuged at 1500 rpm for 5 min, and the unbound CM-DiI was removed. CM-DiI-labeled MSCs were intratracheally infused into normal Sprague-Dawley (S-D) rats for several days. Rats transplanted with unlabeled MSCs served as a control. The recipient rats were maintained for either 1 week, 4 weeks, or 8 weeks. After that, all the rats were sacrificed, and lung tissue slides were prepared (n = 4 per group). Retention of MSCs was evaluated under the fluorescent microscopy.

The effect of CM-DiI staining on MSCs was measured by flow cytometry. For flow cytometric analysis in rats receiving cell transplants, single cell suspensions were generated from one lung lobe by collagenase/dispase (2 mg/ml) digestion.

### Cigarette smoke-induced rat models and MSC treatment

Adult male Sprague-Dawley (S-D) rats weighing 350 ± 10 g were purchased from the Chinese Academy of Sciences and housed under controlled environmental conditions (25°C, 12 h lighting per day) and fed a commercial laboratory chow and water before the experiments were performed. The local institutional animal care committees approved the animal facilities and protocols. All of the rats were divided into four groups (n = 8 per group): (a) sham-exposed group (Sham), (b) cigarette smoke (CS)-exposed group (CS), (c) sham-exposed with MSC treatment group (Sham + MSCs), (d) CS-exposed with MSC treatment group (CS + MSCs). Rats were exposed to the mainstream smoke of 20 filtered commercial cigarettes (each containing 12 mg of tar and 1.25 mg of nicotine; DaQiaMen, Shanghai, China) per day, 5 days per week for 12 weeks in an inhalation box (50 × 40 × 30 cm) as described in our previous study^15^. Sham-exposed animals inhaled clean room air only. Following exposure to cigarette smoke for 7 weeks, rats were anesthetized with pentobarbital (50 mg/kg) and were intratracheally infused with 6 × 10^6^ MSCs suspended in 0.15 ml of PBS or an equal volume of serum-free DMEM twice per week for 5 weeks. At week 16, four weeks after the cessation of smoke exposure, the rats were anesthetized and sacrificed for sample collection.

### Airway inflammation assay and histological analysis

The superior lobe of the right lung was ligated and prepared for histological and morphological analysis. BAL was performed by intratracheal insertion of a catheter and lavage with 5 ml of cold PBS. The fluid was retrieved by gentle aspiration, and this procedure was repeated five times. The BAL fluid was pooled and centrifuged (400 g, 10 min). The supernatants were collected for ELISA, and the cells were harvested for flow cytometry. Blood was collected, and serum was isolated. ELISA was performed to detect the inflammation cytokines, such as PGE2, IL-10, and IL-6.

Next, the lobe of the right lung was removed and immersed in a fixative solution containing 4% paraformaldehyde in 0.1 M PBS (pH 7.0) for 24 h. The samples were processed in preparation for paraffin embedding, and 5-μm-thick sections were cut. Some sections were stained with hematoxylin and eosin (H&E), and others were sequentially subjected to immunostaining analysis. After deparaffinization, the sections were sequentially treated with 1% H_2_O_2_ for 10 min and rinsed thoroughly with PBS. Sections were blocked with 2% normal blocking serum in PBS at room temperature for 60 min to suppress any nonspecific binding of IgG, followed by incubation with COX-2 (dilution 1:200; abcam, Cambridge, UK), or CD68 (dilution 1:100; abcam, Cambridge, UK). All of the slides were evaluated by microscopy. The remaining lung tissues were stored at −80°C until further use.

### Flow cytometry

MSCs were identified using anti-CD11b/c, anti-CD45, anti-CD29 and anti-CD90 antibodies. The cells collected from BAL fluid were stained with anti-CD68 FITC and anti-IL-10 PE (eBioscience, San Diego, CA, USA), as well as isotype controls. After incubation for 30 min at 4°C in the dark, the cells were washed three times with 10 volumes of PBS and centrifuged at 300 g for 5 min. Next, the cells were resuspended in PBS supplemented with 1% FBS and 0.025% sodium azide and analyzed using flow cytometry (BD Biosciences, San Diego, CA). The acquired FACS data were analyzed using FCS Express V4 (BD Biosciences, San Diego, CA). The data were acquired from four independent experiments.

### CSE preparation

Cigarette smoke extract (CSE) was prepared as previously described^24^,^41^. Briefly, 200 ml of cigarette smoke (containing 12 mg of tar and 1.25 mg of nicotine per cigarette; Da Qianmen, Shanghai, China) drawn into a 50-ml plastic syringe through a three-way stopcock was mixed with 20 ml of DMEM by vigorous shaking. Next, the solution was filtered through a 0.22-μm filter and considered a 100% CSE solution. One cigarette was used per 20 ml of CSE, and CSE was prepared no more than 30 min before use in experiments.

### Macrophage stimulation and macrophage-mesenchymal stem cell co-culture experiments

The NR8338 macrophage cell line was obtained from American Type Culture Collection (ATCC, Rockville, MD, USA) and grown to confluence in Dulbecco's modified Eagle's medium (DMEM) containing L-glutamine (2 mM), penicillin (100 IU/ml), streptomycin (100 μg/ml), and 10% fetal calf serum (FBS) at 37°C in a 5% CO_2_ humidified incubator. Macrophages were plated in 6-well plates at a concentration of 1 × 10^6^ cells in 2.6 ml per well. After overnight incubation, macrophages were resuspended in serum-free DMEM and treated with CSE solution or DMEM as a control. MSCs were also cultured in complete medium and transferred to serum-free DMEM until the day of the experiments.

In vitro MSC migration assays were performed using a Transwell system (Corning, Lowell, MA, USA). Briefly, macrophages were added to a 6-well plate, on top of which 8-μm-pore-size Transwells (Corning, Lowell, MA, USA) were placed. MSCs were applied to the top chamber and then incubated for 12 h at 37°C with 5% CO_2_. MSC migration was determined using 0.2% crystal violet staining.

For the co-culture experiments, we placed the two cell populations together in 6-well plates. Macrophages and MSCs were co-cultured with or without CSE solution for 12 h to 48 h at a final concentration of 5–20%. Next, macrophages were collected at 12 h, 24 h and 48 h after the stimulation and subjected to western blot analyses. The supernatants were collected and stored at −20°C until further use for ELISA.

Some CS-exposed macrophages were pretreated with SB203580 (5 μM), U0126 (10 μM), SP600125 (35 μM), or DMSO and cultured with or without MSCs. COX-2 levels in macrophages were determined using western blot analysis.

### 3-(4, 5-Dimethylthiazol-2-yl)-2, 5-diphenyltetrazolium bromide (MTT) assay

For macrophages and MSC co-culture experiments, some macrophages were collected for cell viability assays. Macrophages were treated with CSE solution at a final concentration of 5% to 20% and cultured for 24 h to 72 h, respectively. After co-culture with MSCs, macrophage cells were transferred to a 96-well plate at a density of 3 × 10^3^ cells in 100 μl per well. The MTT assay was performed to measure macrophage cell survival. Cells were stained with 20 μl of sterile MTT dye (Sigma, 5 mg/ml) at 37°C for 4 h followed by removal of the culture medium and thorough mixing with 150 μl of dimethylsulfoxide (DMSO) for 10 min. Spectrometric absorbance was measured at 490 nm. In addition, the cell survival after exposure to the MAPK inhibitors was also performed using the MTT assay. Macrophages were exposed to control medium or medium containing different concentrations of SB203580, U0126, SP600125 (from 5 μM to 50 μM) (Santa Cruz Biotechnology, Dallas, Texas, USA) and anisomycin (from 0.1 μg/ml to 10.0 μg/ml) (abcam, Cambridge, UK). Four independent experiments were performed, and data were presented as the mean ± SEM.

### RNA isolation, cDNA synthesis, and quantitative PCR analysis

Lung tissues reserved previously and macrophages were prepared and subjected to RNA isolation using TRIzol Reagent (Invitrogen Life Technologies), followed by reverse transcription to cDNA. cDNA was synthesized using Prime Script RT Master Mix (Takara Biotechnology, Dalian, China) with all the reagents required for cDNA synthesis, according to the manufacturer's instructions. Quantitative PCR was performed to measure the mRNA levels of COX-2, IL-10, IL-6, and iNOS using SYBR Green Ex Taq™ (Takara) using an ABI 7500 real-time PCR system (Applied Biosystems, Foster City, CA). The PCR protocol consisted of 95°C for 30 sec, followed by 40 cycles of 95°C for 5 sec and 60°C for 34 sec, with a final dissociation. The relative transcript levels were calculated using the 2^−ΔΔ^CT method, in which ^Δ^CT is the difference between the threshold cycle for the gene of interest and that for GAPDH, and the results were normalized to untreated controls. All of the gene sequences were searched using GenBank, and primers were devised (Primer premier 6.0) and synthesized (Invitrogen). The primers were as follows: COX-2, 5′-ACACGGACTTGCTCACTTTGTT-3′ (Forward), 5′-TGGTATT-TCATCTCTCTGCTCTGG-3′ (Reverse); IL-10, 5′-CAGACCCACATGCTCCGAGA-3′ (Forward), 5′-CAAGGCTTGGCAACCCAAGTA-3′ (Reverse); IL-6, 5′-CCACTC-ACAAGTCGGAGGCTTA-3′ (Forward), 5′-GTGCATCATCGCTGTTCATACAATC-3′ (Reverse); iNOS, 5′-TGAGGGGTTTGGAGAGACAGA-3′ (Forward), 5′-CGGG-AGGGAAGGGAGAATAG-3′ (Reverse); GAPDH, 5′-GGCACAGTCAAGGCTGA-GAATG-3′ (Forward), 5′-ATGGTGGTGAAGACGCCAGTA-3′ (Reverse).

### Western blot analysis

Tissues and macrophage cells were harvested and lysed in lysis buffer (10 mM Tris-HCl, pH 7.4, 150 mM NaCl, 0.5% Nonidet P (NP)-40, 1 mM EDTA, 1 mM Na_3_ VO_4_, and 1 mM PMSF) (Pierce, Rockford, IL, USA) containing protease and phosphatase inhibitors. The protein concentration of each sample was determined by the BCA protein assay. Protein extracts made from the lung tissues or macrophages were then subjected to standard sodium dodecyl sulfate-polyacrylamide gel electrophoresis (SDS-PAGE) and transferred to polyvinylidene fluoride (PVDF) membranes. After incubation with a blocking buffer [5% non-fat milk in 10 mM Tris-HCl, pH 7.5, 100 mM NaCl, 0.1% (w/v) Tween-20] for 2 h, the membranes were incubated with antibody against COX-2 (1:1000 dilution), phospho-ERK1/2 (1:1,000 dilution), phospho-p38 (1:1,000 dilution), phospho-JNK (1:1,000 dilution), total ERK1/2 (1:1,000 dilution), p38 (1:1,000 dilution), JNK (1:1,000 dilution) (Cell Signaling Technology, Boston, MA) and GAPDH (1:1,000 dilution) (abcam, Cambridge, UK) overnight at 4°C, followed by a 1 h incubation with the appropriate secondary antibody conjugated to HRP. Detection was performed by enzyme-linked chemiluminescence (ECL, Millipore, MA, USA). The relative quantities of proteins in western blotting were determined by scanning densitometry (ChemiDoc XRS+ Systems, Bio-Rad Laboratories, Inc.) and analyzed using Image Lab 5.0 Software (Bio-Rad Laboratories, Inc.). GAPDH was used as the internal control for protein loading.

### Cytokine measurement by ELISA

The cytokine levels in the BAL fluid, serum and cell culture supernatants were measured by ELISA. PGE2, IL-10, and IL-6 (R&D Systems, Minneapolis, MN). ELISAs were performed according to the manufacturer's directions.

### Statistical analysis

Approximately 5 rats in each group were prepared for histological assay and western blot analysis. Four independent experiments were performed at least for each in vitro co-culture experiments. Statistical analysis was performed using Graphpad Prism Software (San Diego, CA). The results were expressed as the mean ± SEM. One-way ANOVA was used to compare data between groups. The Mann-Whitney *U* test was used for nonparametric analysis. For analysis of the inflammation scores, a nonparametric, Kruskal-Wallis rank-sum test was performed. The *p* values for significance were set to 0.05 for all tests.

## Author Contributions

W.G. designed project, performed experiments, interpreted data and wrote manuscript; L.S. performed experiments, interpreted data; X.M.L. performed PCR and Western blot analysis, D.W. helped to draft and critically revised the manuscript, X.J.G. designed project and performed experiments; W.G.X. designed and supervised project, interpreted data and wrote manuscript. All authors reviewed the manuscript.

## Supplementary Material

Supplementary Information Mesenchymal stem cells alleviate airway inflammation and emphysema in COPD through down-regulation of cyclooxygenase-2 via p38 and ERK MAPK pathways
