# Supplementary material for: Mesenchymal stem cells alleviate airway inflammation and emphysema in COPD through down-regulation of cyclooxygenase-2 via p38 and ERK MAPK pathways
Source: Sci Rep. 2015 Mar 4;5:8733. doi: 10.1038/srep08733 (PMC4348625; doi:10.1038/srep08733)

Mesenchymal stem cells alleviate airway inflammation and emphysema in COPD through down-regulation of cyclooxygenase-2 via p38 and ERK MAPK pathways

Wen Gu[1], Lin Song[1], Xiao-Ming Li[1], Di Wang[1], Xue-Jun Guo*[1] and Wei-Guo Xu*[1]

[1]Department of Respiratory Medicine, Xinhua Hospital, School of Medicine, Shanghai Jiaotong University, 1665 KongJiang Road, Shanghai 200092, China.

(* Xue-Jun Guo and Wei-Guo Xu equally contributed to the work)

Correspondence and requests for materials should be addressed to W.G.X. (xuweiguoxinhua@126.com) or X.J.G. (guoxj1964@126.com)

Supplementary Figure Legends

Fig S1. MSC cultivation and characterization. A. MSCs were isolated from rat bone marrow aspirates and transferred to culture plates. MSCs, which had a spindle fibroblast-like appearance, were allowed to proliferate. The cells were plated on day 5 and reached 80% confluence 14 days later. **B**. The rMSCs did not express CD11b/c or CD45 but did express CD29 and CD90 by flow cytometry. Four independent experiments were performed with similar results.

Fig S2. Retention of MSCs was evaluated after transplantation into CS-exposed rat models. A. Lung slides were stained to identify CM-DiI-positive cells using fluorescence microscopy (×100 magnification). **B.** Representative flow cytometry plots show a higher frequency of CM-DiI-positive cells at 1 week after MSC transplantation (1.66%±0.09% cells). The percentage of CM-DiI-positive cells decreased over the following 8 weeks (0.54%±0.04% at 4 weeks and 0.24%±0.033% at 8 weeks).

Fig S3. Relief of airway inflammation and emphysema by MSC administration in CS-exposed rat models. The CS-exposed rat models were established, and rats were anesthetized with pentobarbital (50 mg/kg) and intratracheally infused with 6×10^6 MSCs suspended in 0.15 ml of PBS twice per week for 5 weeks beginning at the 7th week. **A** and **C.** Lung sections were subjected to H&E staining. The inflammatory cells infiltrated into the peribronchial and perivascular lung tissues in the CS-exposed group, and the airway inflammation was ameliorated after MSC administration (n=5 per group, ×100 magnification). Inflammation scores were presented as the mean±SEM of 5 rats/group. MSC treatment decreased CS-induced peribronchial and perivascular inflammation. *significant difference (P <0.05) between the CS and CS+MSCs groups. **B** and **D**. Morphometric analysis of the mean linear intercept (MLI) was used to assess the air space enlargement. The MLI increased in the CS-exposed group and decreased after MSC administration. Data represent the mean±SEM. **significant difference (P <0.01) between the CS and Sham groups. *significant difference (P <0.05) between the CS and CS+MSCs groups. **E-G.** The proinflammatory factors PGE2, IL-10, and IL-6 in the BAL and serum were detected by ELISA. The BAL and serum showed a significant increase in PGE2 and IL-6 in the CS group compared with the levels in the Sham group. A significant decrease in PGE2 and IL-6 was observed in the CS+MSCs group compared with the levels in the CS group. However, IL-10 levels were decreased in the CS group and were increased in the CS+MSCs group in both the BAL and serum. Data represent the mean±SEM, n=5. **significant difference (P <0.01) and *significant difference (P <0.05) between the CS and CS+MSCs groups.

Supplementary Fig S1

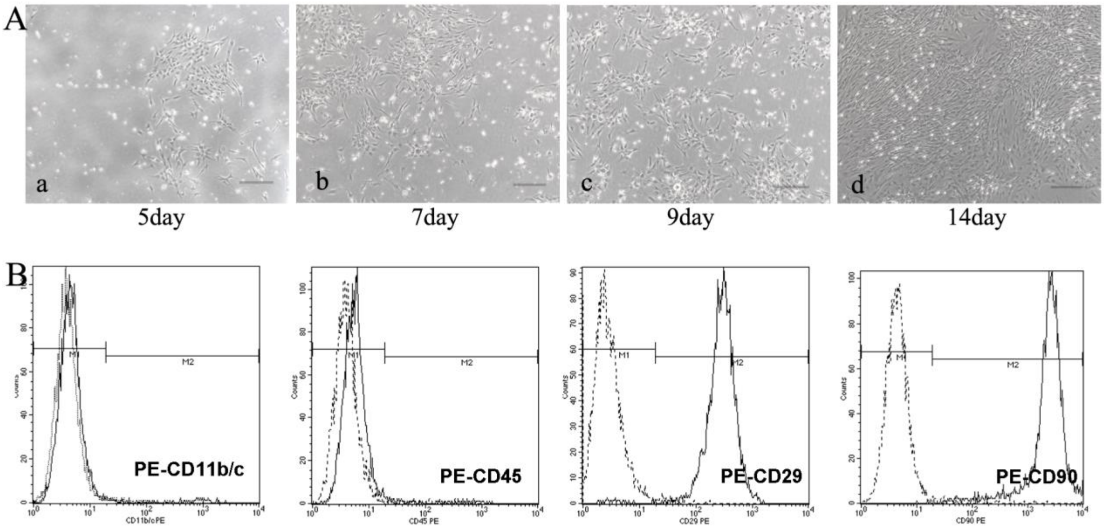

Supplementary Fig S2

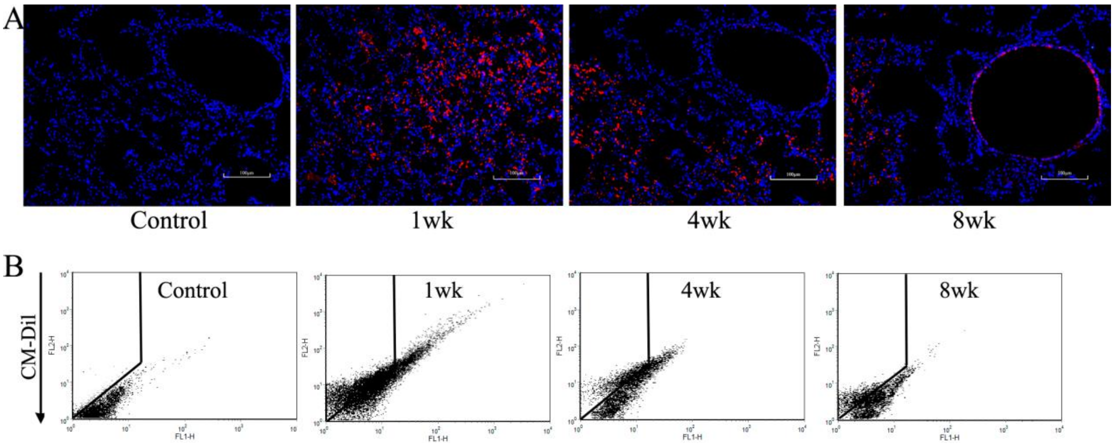

Supplementary Fig S3

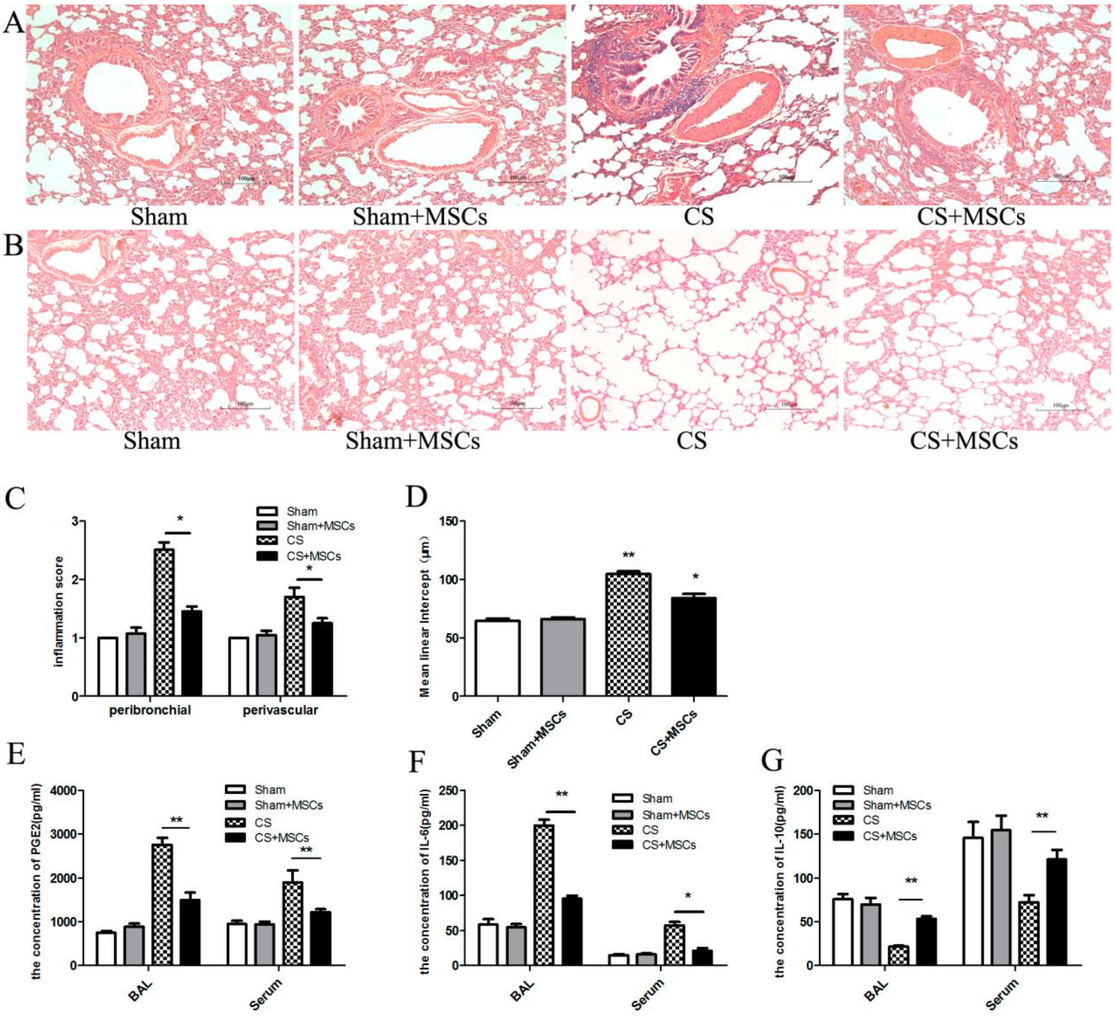

Supplementary Figure S4

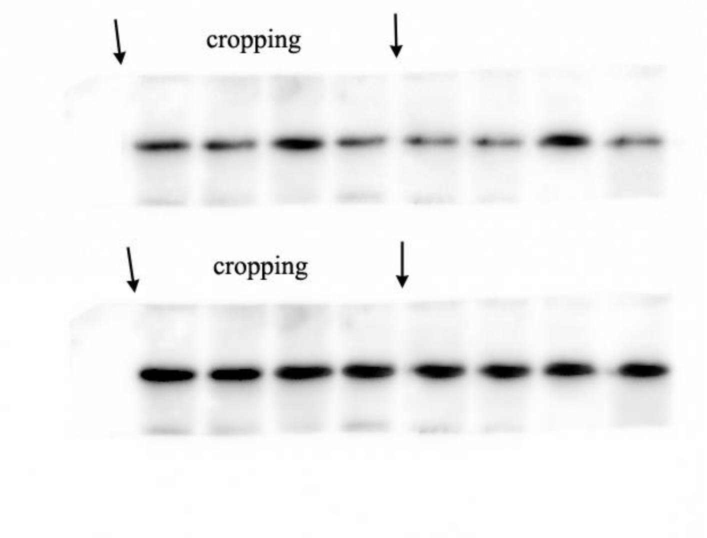

Supplementary Figure S5

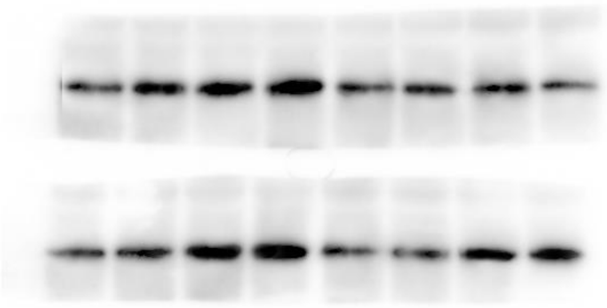

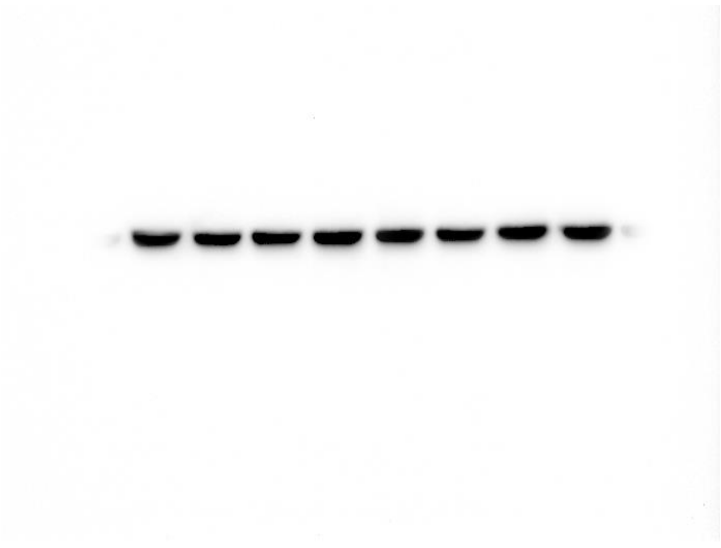

Supplementary Figure S6

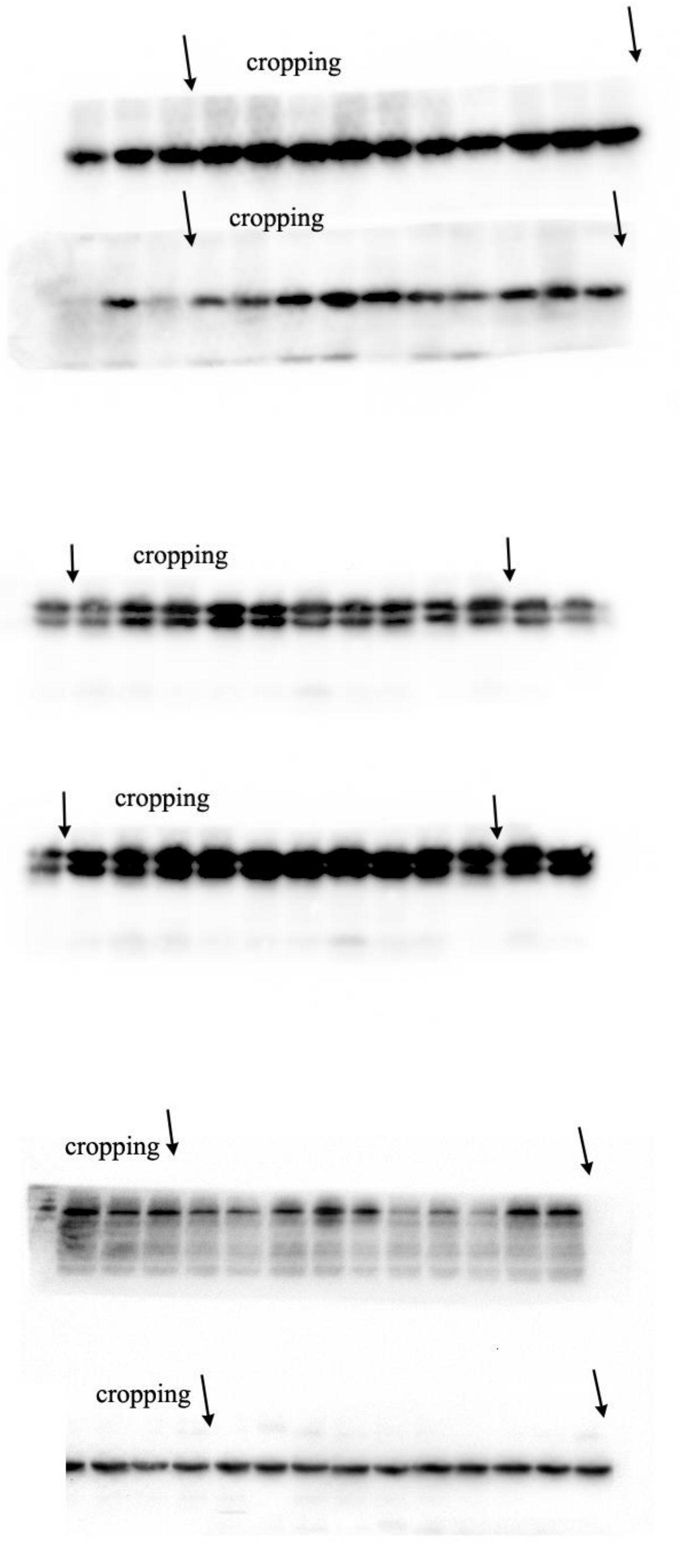

Supplementary Figure S7

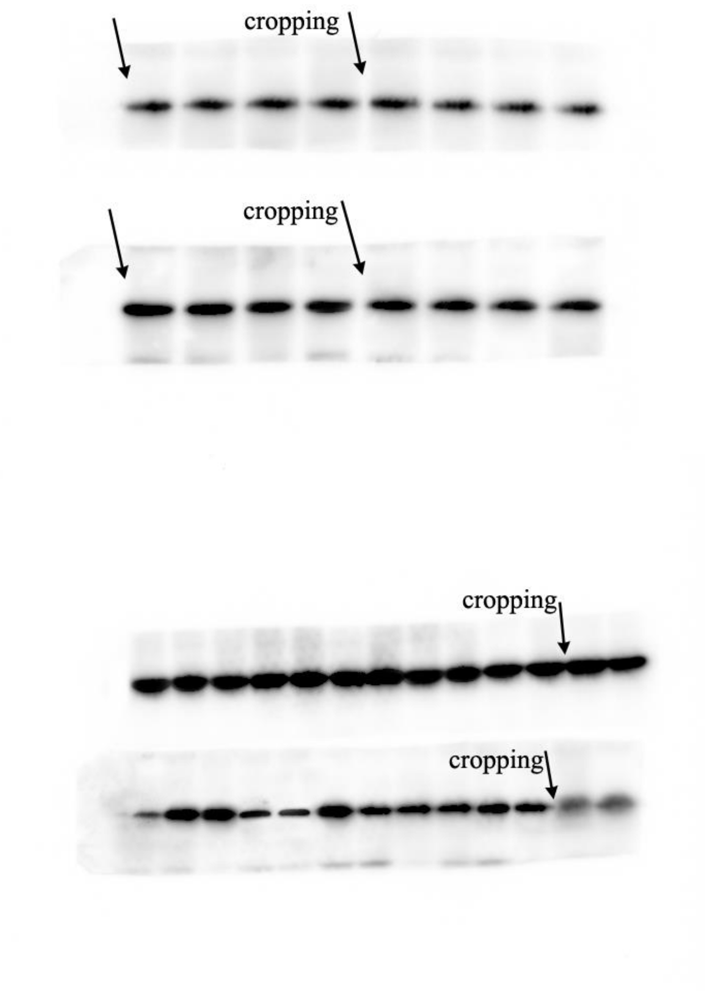

Supplementary Figure S8

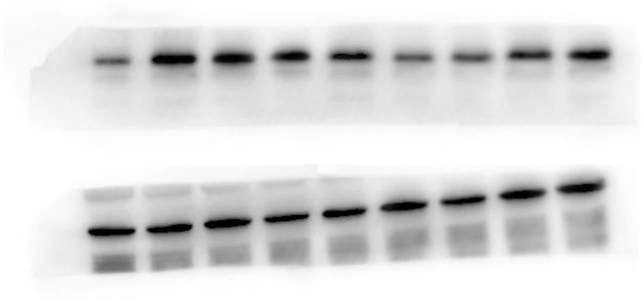

Supplement: Supplementary Information — Mesenchymal stem cells alleviate airway inflammation and emphysema in COPD through down-regulation of cyclooxygenase-2 via p38 and ERK MAPK pathways [file srep08733-s1.pdf]
